# Supplementary material for: Prognostic Factors in Epithelioid Hemangioendothelioma: Analysis of a Nationwide Molecularly/Immunohistochemically Confirmed Cohort of 57 Cases
Source: Cancers (Basel). 2023 Jun 23;15(13):3304. doi: 10.3390/cancers15133304 (PMC10340645; doi:10.3390/cancers15133304)
Supplement: Supplementary file 1 [file cancers-15-03304-s001.zip › cancers-2456882-supplementary.pdf]

Supplementary Table S1. Details about systemic treatment in patients with either lymph node metastases or multiple metastases.

Case	Systemic therapy	Details
3	No	
10	No	
21	Yes	Pacliataxel, doxorubicin
22	No	
27	No	
29	Yes	Interferon-alfa
37	Yes	Pacliataxel
40	Yes	Pacliataxel
35	Yes	Pacliataxel
34	No	
30	Yes	Docetaxel
31	Yes	Paclitaxel
33	No	
38	Yes	Pacliataxel
39	No	
43	No	
45	Yes	Adriamycin
46	Yes	Curative chemotherapy, unspecified
47	Yes	Unknown
48	No	
50	No	
51	Yes	Cyclophosphamide
54	No	
